# Respiratory effects of lung recruitment maneuvers depend on the recruitment-to-inflation ratio in patients with COVID-19-related acute respiratory distress syndrome

**DOI:** 10.1186/s13054-021-03876-z

**Published:** 2022-01-04

**Authors:** Yoann Zerbib, Alexis Lambour, Julien Maizel, Loay Kontar, Bertrand De Cagny, Thierry Soupison, Thomas Bradier, Michel Slama, Clément Brault

**Affiliations:** Intensive Care Department, CHU Amiens-Picardie, 1 Rue du Professeur Christian Cabrol, 80000 Amiens, France

**Keywords:** Acute respiratory distress syndrome, Recruitment maneuver, Recruitability, Mechanical ventilation, Respiratory mechanics

## Abstract

**Background:**

In the context of acute respiratory distress syndrome (ARDS), the response to lung recruitment maneuvers (LRMs) varies considerably from one patient to another and so is difficult to predict. The aim of the study was to determine whether or not the recruitment-to-inflation (R/I) ratio could differentiate between patients according to the change in lung mechanics during the LRM.

**Methods:**

We evaluated the changes in gas exchange and respiratory mechanics induced by a stepwise LRM at a constant driving pressure of 15 cmH_2_O during pressure-controlled ventilation. We assessed lung recruitability by measuring the R/I ratio. Patients were dichotomized with regard to the median R/I ratio.

**Results:**

We included 30 patients with moderate-to-severe ARDS and a median [interquartile range] R/I ratio of 0.62 [0.42–0.83]. After the LRM, patients with high recruitability (R/I ratio ≥ 0.62) presented an improvement in the P_a_O_2_/F_i_O_2_ ratio, due to significant increase in respiratory system compliance (33 [27–42] vs. 42 [35–60] mL/cmH_2_O; *p* < 0.001). In low recruitability patients (R/I < 0.62), the increase in P_a_O_2_/F_i_O_2_ ratio was associated with a significant decrease in pulse pressure as a surrogate of cardiac output (70 [55–85] vs. 50 [51–67] mmHg; *p* = 0.01) but not with a significant change in respiratory system compliance (33 [24–47] vs. 35 [25–47] mL/cmH_2_O; *p* = 0.74).

**Conclusion:**

After the LRM, patients with high recruitability presented a significant increase in respiratory system compliance (indicating a gain in ventilated area), while those with low recruitability presented a decrease in pulse pressure suggesting a drop in cardiac output and therefore in intrapulmonary shunt.

**Supplementary Information:**

The online version contains supplementary material available at 10.1186/s13054-021-03876-z.

## Introduction

Acute respiratory distress syndrome (ARDS) is characterized by increased pulmonary vascular permeability, alveolar edema, and loss of aerated lung. Severe hypoxemia and impaired pulmonary compliance are the main clinical features of ARDS. We estimate that prevalence of ARDS (according to the 2012 Berlin definition) is 10% in the ICU and 40% among ventilated patients [1, 2]. Over the last few decades, optimized ventilator management (with a reduction in tidal volume), the use of a higher positive end-expiratory pressure (PEEP), and prone positioning have enabled a reduction in the mortality rate [3–8]. In addition to PEEP, lung recruitment maneuvers (LRMs) might beneficial in routine practice; the transpulmonary pressure is transiently increased, in order to fully recruit collapsed alveoli and improve oxygenation [9]. In ARDS, the response to positive pressure (PEEP or LRM) is hard to predict because it depends on lung recruitability and varies considerably from one patient to another. Moreover, increasing the PEEP or performing an LRM can be harmful—especially in patients with low recruitability. Indeed, applying an excessive PEEP may induce lung overdistention and thus left and/or right cardiac dysfunction. Therefore, predicting the response and tolerance to positive pressure is a major challenge for clinicians. There are no simple, accurate tools for the clinical assessment of lung recruitability. Computed tomography, ultrasound and electrical impedance tomography are promising but are complex to apply at the bedside. Recently, a single-breath maneuver with measurement of the recruitment-to-inflation (R/I) ratio has been developed to (i) evaluate the potential for lung recruitment and (ii) identify patients who could benefit from the application of positive pressure [10]. Several studies used the R/I ratio to assess lung recruitability in COVID-19-related ARDS [11–13]. Results suggested a high lung recruitability, with a great variability between patients and studies. We supposed that LRMs lead to different effects on respiratory mechanisms, gas exchange and hemodynamics, depending on the potential for lung recruitment.

The objective of the present study was to determine whether or not the R/I ratio could differentiate between patients according to the change in lung mechanics during the LRM.

## Methods

Study population: Patients undergoing mechanical ventilation for COVID-19-related ARDS in the intensive care department at Amiens University Medical Center (Amiens, France) between March 1st and November 30th, 2020. The study was approved by the local institutional review board (CPP Nord-Ouest II, Amiens, France; reference: CEERNI 110). All patients met the Berlin definition for ARDS and were positive for SARS-CoV-2 RNA in a real-time PCR assay of a nasopharyngeal swab. We excluded patients with an arterial oxygen partial pressure (P_a_O_2_) to fractional inspired oxygen (F_i_O_2_) ratio above 150 mmHg and those with pneumothorax, pneumomediastinum, or hemodynamic instability (defined as an increase in vasoactive drug levels in the previous six hours).

Ventilation strategies: All patients were ventilated in volume-control mode using V500 (Drager, Lübeck, Germany) or Servo i (Maquet, Solna, Sweden) systems. Sedation and analgesia were achieved with continuous intravenous infusion of midazolam or propofol. Neuromuscular blockade was obtained through the continuous intravenous infusion of cisatracurium. The tidal volume was set to 6 mL per kilogram of predicted body weight, and the pressure plateau was kept below 28–30 cmH_2_O. The F_i_O_2_ level was adjusted to achieve peripheral oxygen saturation (S_p_O_2_) of 88–92%.

Recruitment-to-inflation ratio: Given that an airway opening pressure (AOP) can prompt the misinterpretation of respiratory mechanics data, we detected and measured this variable during a prolonged inhalation with a 5 L/min inspiratory flow. Next, we measured the recruitment-to-inflation (R/I) ratio, as described by Chen et al. [10]. During a single breath, we abruptly decreased the PEEP (from 15 cmH_2_O or the AOP + 10 cmH_2_O to 5 cmH_2_O or the AOP) and measured the induced change in end-expiratory lung volumes (ΔEELV). We calculated the recruited volume (ΔV_rec_) as the difference between the measured ΔEELV and the predicted ΔEELV (i.e., the compliance at low PEEP multiplied by the change in PEEP). The ΔV_rec_ divided by the change in PEEP gave the recruited lung’s compliance (C_rec_). The R/I ratio was defined as the ratio between the C_rec_ and the respiratory system compliance (C_rs_) at low PEEP. The higher the R/I ratio, the more the compliant the recruited lung is, and therefore, the greater the volume recruited compared to the hyperinflated volume. Conversely, the lower the R/I ratio, the higher the risk of overdistention without benefit in terms of recruitment during PEEP increase. High recruitability was defined as an R/I ratio above the median for the population [10].

Lung recruitment protocol: We performed a stepwise LRM in pressure-control mode and with a driving pressure of 15 cmH_2_O (see in the Additional file 1: Fig. E1). Starting at 20 cmH_2_O, the PEEP was increased in 5 cmH_2_O steps to 40 cmH_2_O, with each step lasting 2 min. The safety endpoints for interruption of the LRM were a S_p_O_2_ under 88% or a decrease of more than 20% in the heart rate or mean arterial pressure. The LRM was immediately followed by a decremental PEEP titration (2 cmH_2_O every 2 min) from 25 cmH_2_O until the PEEP level chosen by the clinician before the LRM. Data were collected just before and then after the LRM, at the same PEEP level. Another LRM was then performed, and the PEEP was reset to the optimal level (i.e., a lower PEEP for the highest S_p_O_2_). To avoid interference with the immediate effect of LRM, the P_a_O_2_/F_i_O_2_ ratio and the ventilator ratio (VR) were measured in the 3 h following the LRM, at the optimal PEEP level. The esophageal pressure measurement and VR calculation are described in the Additional file 1 (see Method E1 and E2).

Statistical analysis: Data were quoted as the median [interquartile range (IQR)] or the frequency (percentage), as appropriate. For comparisons of categorical variables, we used a chi-square test or Fisher’s exact test, as appropriate. For normally and non-normally distributed continuous variables, we used Student’s *t* test and the Mann–Whitney test, respectively. The correlation between the R/I ratio and the change in C_rs_ was assessed with Spearman’s rho. All statistical analyses were performed using GraphPad Prism software (version 8.0.0, GraphPad Software, San Diego, CA, USA). The threshold for statistical significance was set to *p* < 0.05.

## Results

Between March and December 2020, a total of 41 patients with COVID-19-related ARDS met the inclusion criteria (see Additional file 1: Fig. E2). Eleven patients were excluded because of a P_a_O_2_/F_i_O_2_ ratio above 150 (*n* = 9), or pneumodiastinum (*n* = 2). Our final analysis therefore covered 30 patients, whose main characteristics are summarized in Table 1. The median [IQR] R/I ratio was 0.62 [0.42–0.83]. Details of LRM tolerability are given in the Additional file 1 (see Table E1).

**Table 1:** General characteristics of the study population

Parameters	*n* (%) Median [IQR]
Demographic data	
Sex, male	23 (77)
Age, years	67 [58–74]
BMI, kg/m^2^	33 [29–38]
Computed tomography findings	
Diffuse pattern	24 (100)
Interstitial syndrome	24 (100)
Alveolar consolidation	15 (63)
Lung damage extension, %	50 [28–71]
Gas exchange	
VR	2.07 [1.61–2.51]
P_a_O_2_/F_i_O_2_, mmHg	105 [86–133]
Severe ARDS	12 (40)
Respiratory mechanics	
Tidal volume, ml/kg PBW	6.1 [5.9–6.3]
Respiratory rate, breaths/min	28 [25–30]
Plateau pressure, cmH_2_O	26 [23–28]
PEEP applied, cmH_2_O	13 [8–16]
C_rs_, mL/cmH_2_O	33 [26–42]
Airway closure	15 (50)
R/I ratio	0.62 [0.42–0.83]
Death in the ICU	14 (46)

AOP: airway opening pressure, ARDS: acute respiratory distress syndrome, BMI: body mass index, C_rs_: respiratory system compliance, ICU: intensive care unit, IQR: interquartile range, P_a_O_2_/F_i_O_2_: partial pressure of oxygen to inspired oxygen fraction, PEEP: positive end-expiratory pressure, R/I: recruitment-to-inflation, VR: ventilator ratio

### Respiratory system compliance

**Fig. 1:**
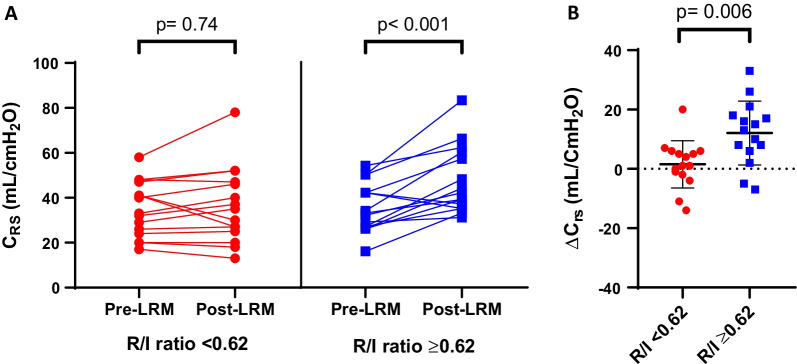
Effects of the LRM on respiratory system compliance, as a function of the patients’ lung recruitability. **A** pre- and post-LRM C_rs_ in patients with low lung recruitability (red circles) or high lung recruitability (blue squares). **B** The change in C_rs_ induced by the LRM in patients with low lung recruitability (red circles) or high lung recruitability (blue squares). Low and high lung recruitability was defined, respectively, as an R/I ratio below or above the median value for the cohort (0.62). C_rs_: respiratory system compliance, ns: nonsignificant, R/I: recruitment-to-inflation ratio

In patients with low lung recruitability, the C_rs_ was not significantly modified by the LRM (33 [24–47] vs. 35 [25–47] mL/cmH_2_O; *p* = 0.74). Conversely, in patients with high lung recruitability, the C_rs_ increased significantly after the LRM (33 [27–42] vs. 42 [35–60] mL/cmH_2_O; *p* < 0.001) (Table 2 and Fig. 1A). The absolute change in C_rs_ during the LRM was significantly higher in patients with high lung recruitability than in patients with low lung recruitability (13 [6–18] vs. 1 [− 2 to 6] mL/cmH_2_O; *p* = 0.006) (Fig. 1B). The area under the curve for R/I ratio as a predictor of lung recruitment after a maximal LRM was 0.80 (95% CI, 0.61–0.99; *p* = 0.02) (see Additional file 1: Fig. E3). An R/I ratio > 0.62 predicted recruitment after the LRM with a sensitivity of 83% and a specificity of 58%. An R/I ratio > 0.8 predicted recruitment with a sensitivity of 37%, a specificity of 100%, and positive and negative predictive values of 100% and 30%, respectively.

**Table 2:** Respiratory and hemodynamic parameters before and after the LRM, as a function of the patients’ lung recruitability

Parameters	R/I < 0.62 *n* = 15			R/I ≥ 0.62 *n* = 15		
	Pre-LRM	Post-LRM	*p* value	Pre-LRM	Post-LRM	*p* value
C_rs_, mL/ cmH_2_O	33 [24–47]	35 [25–47]	0.74	33 [27–42]	42 [35–60]	< 0.001
P_L,EE_, cmH_2_O	− 2 [− 6 to 2]	2 [− 1 to 5]	0.002	− 4 [− 6 to 2]	1 [− 2 to 3]	< 0.001
P_L,EE_ < 0 cmH_2_O, *n* (%)	8 (53)	4 (27)	0.26	9 (60)	4 (27)	0.14
P_L,EI_, cmH_2_O	12 [9–17]	14 [10–17]	> 0.99	15 [11–18]	16 [11–19]	0.96
P_L,EI_ ≥ 25 cmH_2_O, *n* (%)	0 (0)	0 (0)	> 0.99	0 (0)	0 (0)	> 0.99
P_a_O_2_/F_i_O_2_, mmHg	106 [101–132]	186 [128–192]	0.01	99 [73–131]	106 [98–151]	0.048
P_a_CO_2_, mmHg	46 [44–56]	46 [43–56]	0.66	42 [36–48]	46 [38–53]	0.16
VR	2.1 [1.9–2.5]	2.1 [1.8–2.6]	0.88	2.0 [1.5–2.6]	2.0 [1.6–2.4]	0.91
PP, mmHg	70 [55–85]	50 [51–67]	0.01	61 [43–70]	54 [46–70]	0.51
HR, beats/min	96 [73–105]	92 [68–112]	0.56	80 [68–105]	88 [64–96]	0.74

C_rs_: respiratory system compliance, HR: heart rate, LRM: lung recruitment maneuver, P_a_O_2_/F_i_O_2_: partial pressure of oxygen to inspired oxygen fraction, P_L,EE_: transpulmonary pressure at end-expiration, P_L,EI_: transpulmonary pressure at end-inspiration, PP: pulse pressure, VR: ventilator ratio

### Gas exchange

**Fig. 2:**
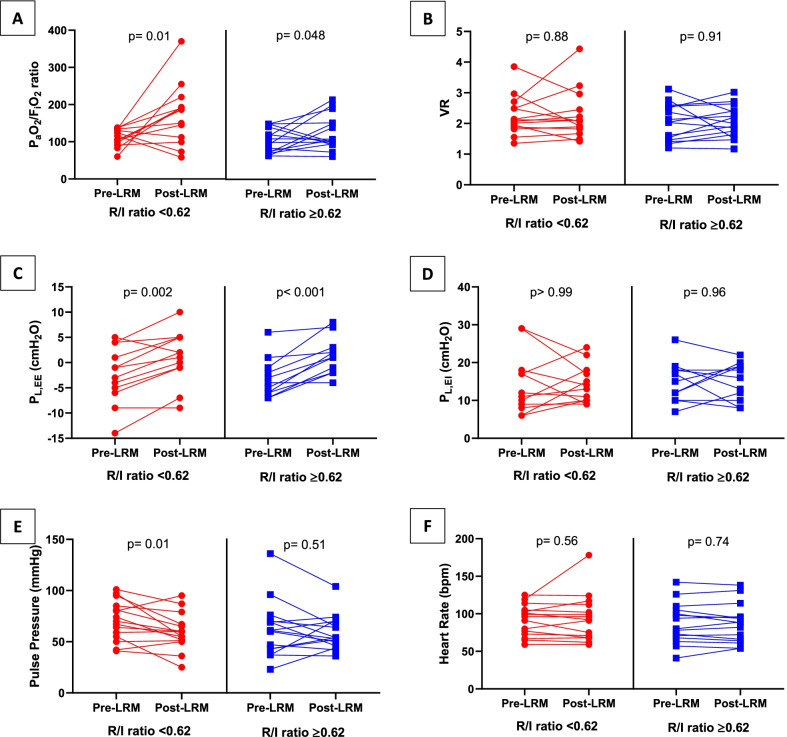
Effects of the LRM on respiratory mechanics and gas exchanges, as a function of the patients’ lung recruitability. Effects of LRM on P_a_O_2_/F_i_O_2_ and ventilator ratio (**A**, **B**), end-expiratory and end-inspiratory transpulmonary pressures (**C**, **D**), and pulse pressure and heart rate (**E**, **F**). Patients with low lung recruitability (red circles) and high lung recruitability (blue squares) were defined by an R/I below or above the median value for the cohort (0.62). HR: heart rate, P_a_O_2_/F_i_O_2_: partial pressure of oxygen to inspired oxygen fraction, P_L,EE_: transpulmonary pressure at end-expiration, P_L,EI_: transpulmonary pressure at end-inspiration, PP: pulse pressure, R/I: recruitment-to-inflation ratio, VR: ventilator ratio

The P_a_O_2_/F_i_O_2_ ratio increased significantly after LRM in patients with low lung recruitability (106 [101–132] vs. 186 [128–192] mmHg; *p* = 0.01) and high lung recruitability (99 [73–131] vs. 106 [98–151] mmHg; *p* = 0.048). In contrast, neither the partial pressure of carbon dioxide in arterial blood nor the VR was not modified by the LRM (Table 2, Fig. 2A, B).

### Transpulmonary pressure

The end-expiratory transpulmonary pressure (P_L,EE_) increased significantly during the LRM in both patients with low lung recruitability (− 2 [− 6 to 2] vs. 2 [− 1 to 5] cmH_2_O; *p* = 0.002) and high lung recruitability (− 4 [− 6 to 2] vs. 1 [− 2 to 3] cmH_2_O; *p* < 0.001). After the LRM, the P_L,EE_ stayed negative in four (27%) patients with low recruitability and four (27%) patients with high recruitability. Results concerning changes in C_rs_ and P_a_O_2_/F_i_O_2_ ratio in patients with a positive P_L,EE_ after the LRM are shown in Additional file 1 (See Table E2).

In contrast, the end-inspiratory transpulmonary pressure (P_L,EI_) was not modified by the LRM in patients with low lung recruitability (12 [9–17] vs. 14 [10–17] cmH_2_O; *p* > 0.99) or those with high lung recruitability (15 [11–18] vs. 16 [11–19] cmH_2_O; *p* = 0.96). None of the patients in either group had a P_L,EI_ > 25 cmH_2_O after the LRM (*p* > 0.99) (Table 2, Fig. 2C, D).

### Hemodynamic parameters

Patients with low and high lung recruitability differed in their hemodynamic tolerability of the LRM. In patients with low lung recruitability, the LRM induced a significant decrease in systolic but not diastolic blood pressure, leading to a significant decrease in the pulse pressure (70 [55–85] vs. 50 [51–67] mmHg; *p* = 0.01). In patients with high lung recruitability, the pulse pressure was not modified by the LRM (61 [43–70] vs. 54 [46–70] mmHg; *p* = 0.51). Finally, the heart rate remained stable during the LRM (Table 2 and Additional file 1: E3, Fig. 2E, F).

## Discussion

We conducted a physiology-based study of gas exchanges, lung mechanics, and hemodynamic status. We observed a significant increase in the P_a_O_2_/F_i_O_2_ ratio during the LRM in patients with low lung recruitability and in those with high lung recruitability (especially when we focused on patients with positive P_L,EE_ after LRM, see Table E2). However, the mechanisms behind this improvement in oxygenation depend on each patient’s potential for alveolar recruitment, as measured by the R/I ratio.

The severe impairment of oxygenation in ARDS is caused by a marked decrease in aerated lung, which leads to ventilation-perfusion mismatches and an increase in the shunt fraction. Mechanical ventilation with sufficient PEEP is intended to recruit alveoli and prevent their collapse. Furthermore, LRMs are associated with better oxygenation and without influencing the mortality rate [14–17]. LRMs promote alveolar recruitment by transiently increasing the transpulmonary pressure and reopening non-aerated or poorly aerated alveolar units [18]. Due to lung heterogeneity in ARDS, the LRM can sometimes be associated with hyperinflation of lung parts that are already open (i.e., the “baby lung”) and hemodynamic instability [14]. In 2017, the multicenter ART trial randomized 1010 patients with moderate-to-severe ARDS and found a higher mortality rate in those exposed to LRMs [9]. Consequently, the risk/benefit ratio of LRMs in ARDS is still subject to debate; the latest guidelines suggest that LRMs should be considered for selected patients but do not provide further details [19–21]. Hence, there is a need to identify factors that predict a response to LRMs in patients with ARDS. The severity of ARDS (according to the P_a_O_2_/F_i_O_2_) or the type (pulmonary vs. non-pulmonary) fails to identify LRM responders [9, 22]. Likewise, the LIVE study failed to show a benefit of personalized ventilation and LRMs as a function of the lung morphology on a CT scan (diffuse vs. focal) [23]. The R/I ratio is a new bedside tool that might help to distinguish between patients with low and high lung recruitment potentials [10]. The ratio expresses the relationship between the compliances of recruited lung and ventilated lung at low PEEP; the higher the R/I ratio, the greater the recruited volume compared to the overdistended volume. Conversely, a low R/I ratio is associated with a greater risk of hyperinflation and a lack of benefit in terms of recruitment during the PEEP increase. In our study, the performance of R/I ratio to predict increase in C_rs_ after LRM was promising. More importantly, increase in C_rs_ was predictable in patients with R/I ratio above 0.8 (25% of the study population). These very selected patients with very high lung recruitability based on R/I ratio (> 0.8) might benefit from LRM. Altogether, these results support an individual used of LRM based on R/I ratio and a confirmation in larger studies is needed.

In patients with high lung recruitability, we observed an increase in oxygenation due to a significant increase in C_rs_. We also observed a significant increase in the P_L,EE_. Only four patients with high recruitability still had a negative P_L,EE_ (a marker of derecruitment in dependent zones) after the LRM. Taken as a whole, these results indicate a decrease in non-aerated lung tissue in these patients, which in turn decreased the intrapulmonary shunt (Q_s_/Q_t_) [24]. In patients with low lung recruitability, the increase in P_a_O_2_/F_i_O_2_ was not associated with a significative increase in C_rs_. Conversely, the LRM induced a decrease in C_rs_ in 5 (33%) patients with low lung recruitability. However, we found a significant decrease in pulse pressure—a surrogate of cardiac output. Thus, the increase in oxygenation might be related to a reduction in Q_s_/Q_t_ without an increase in aerated lung tissue [19]. Interestingly, we did not find any signs of overdistention after the LRM because (i) none of the patients had a P_L,EI_ above 25 cmH_2_O, and (ii) the VR (a surrogate marker of dead space) did not change significantly with the LRM. However, the negative cardiovascular impact of LRMs had already been reported—even in the absence of alveolar hyperinflation. By modifying the lung volume and intrathoracic pressure, LRMs decrease venous return (especially in cases with concomitant hypovolemia) and right ventricular (RV) preload and increase the RV afterload. Consequently, the left ventricular preload is reduced, which in turn decreases the cardiac output [25, 26]. Hypotension requiring increased vasopressor use during the procedure occurred in 13% of the patients in the PHARLAP study, while severe hypotension leading to the interruption of LRM occurred in 11% of patients in the ART trial [9, 22]. We can therefore assume that the increase in oxygenation observed in these patients was explained (at least in part) by a reduction in cardiac output and thus a decrease in Q_s_/Q_t_ [24, 27].

PEEP-induced changes in lung aeration as a function of the R/I ratio have not been extensively studied. Our group has used transesophageal echography to demonstrate the significant re-aeration of the lower lobes (where consolidations predominate) in high recruiters only [28]. This is consistent with Stevic et al.’s transthoracic echography study of the lung ultrasound (LUS) aeration score [11]. The LUS aeration score for posterior (dependent) lung regions was greater in high recruiters than in low recruiters. In contrast, there was no intergroup difference in the LUS aeration score for anterior (non-dependent) lung regions. Interestingly, the R/I ratio is also correlated with the response to a move to the prone position—another established method for lung recruitment. Cour et al. found a strong, significant correlation between the R/I ratio and the change in C_rs_ during a move from the supine position to the prone position [12]. This response depended on the R/I ratio, as only high recruiters showed a significant increase in C_rs_, which persisted after repositioning in the supine position.

Our study had several limitations. Firstly, we did not directly assess the effect of LRM on end-expiratory lung volume, cardiac output and intrapulmonary shunt; hence, we cannot confirm the suggested hypotheses. Secondly, various other LRM techniques have been described: sustained continuous positive airway pressure, extended sigh, and pressure-controlled ventilation with progressive increases in PEEP maintaining a pressure driving pressure. The LRMs’ effects might depend on the pressure level reached and/or the duration of exposure. The level of pressure needed to open atelectactic lung cannot be calculated precisely, and 40 cmH_2_O might not suffice [29].

## Conclusion

During an LRM, the mechanisms related to an increase in P_a_O_2_/F_i_O_2_ ratio depend on the potential for lung recruitment. Patients with high lung recruitability presented a significant increase in C_rs_, indicating a gain in ventilated area. Patients with low lung recruitability presented a significant decrease in pulse pressure, suggesting a drop in cardiac output and therefore in intrapulmonary shunt. The R/I ratio (an easy-to-use bedside tool for assessing lung recruitability) might help clinicians to identify patients in whom an LRM will lead to an increase in C_rs_. Further studies are needed to confirm our present findings.

## Supplementary Information

**Additional file 1**. **Method E1**: Esophageal pressure measurement; **Method E2**: Ventilatory ratio calculation; **Table E1**: Lung recruitment maneuver and titration of the optimal positive end-expiratory pressure; **Table E2**: Effects of lung recruitment maneuver according to lung recruitability, in patients with positive end-expiratory transpulmonary pressure; **Table E3**: Hemodynamic changes during lung recruitment maneuver according to lung recruitability; **Figure E1**: Protocol for the lung recruitment maneuver in patient #12; **Figure E2**: Study flow chart; **Figure E3**: The receiver operating characteristic curve for the prediction of lung recruitment after the lung recruitment maneuver based on the recruitment-to-inflation ratio.

## Data Availability

The raw data supporting the conclusions of this article will be made available by the authors, without undue reservation.
