# Supplementary material for: Respiratory effects of lung recruitment maneuvers depend on the recruitment-to-inflation ratio in patients with COVID-19-related acute respiratory distress syndrome
Source: Crit Care. 2022 Jan 4;26:12. doi: 10.1186/s13054-021-03876-z (PMC8727044; doi:10.1186/s13054-021-03876-z)
Supplement: Supplementary file 1 — Additional file 1. Method E1: Esophageal pressure measurement; Method E2: Ventilatory ratio calculation; Table E1: Lung recruitment maneuver and titration of the optimal positive end-expiratory pressure; Table E2: Effects of lung recruitment maneuver according to lung recruitability, in patients with positive end-expiratory transpulmonary pressure; Table E3: Hemodynamic changes during lung recruitment maneuver according to lung recruitability; Figure E1: Protocol for the lung recruitment maneuver in patient #12; Figure E2: Study flow chart; Figure E3: The receiver operating characteristic curve for the prediction of lung recruitment after the lung recruitment maneuver based on the recruitment-to-inflation ratio. [file 13054_2021_3876_MOESM1_ESM.docx]

**Additional file – Method E1**

Esophageal pressure measurement.

An esophageal balloon-catheter (Nutriven®, Sidam, Modena, Italy) was inserted and filled with 3 mL of air to evaluate esophageal pressure (P_oe_), as a surrogate marker of pleural pressure. We used a chest X-ray and a positive pressure occlusion test to check that the balloon was well placed, as described previously (E1). We measured the end-expiratory transpulmonary pressure (PL_EE_, the difference between PEEP and end-expiratory P_oe_) and the end-inspiratory transpulmonary pressure (PL_EI_, the product of the plateau pressure and the ratio of lung and respiratory system elastances) (E2).

E1. Chiumello D, Consonni D, Coppola S, Froio S, Crimella F, Colombo A. The occlusion tests and end-expiratory esophageal pressure: measurements and comparison in controlled and assisted ventilation. Ann Intensive Care. déc 2016;6(1):13.

E2. Yoshida T, Amato MBP, Grieco DL, Chen L, Lima CAS, Roldan R, et al. Esophageal Manometry and Regional Transpulmonary Pressure in Lung Injury. Am J Respir Crit Care Med. 15 avr 2018;197(8):1018‑26.

**Additional file – Method E2**

Ventilatory ratio calculation.

The ventilatory ratio (VR) is an indication of overall efficiency of ventilation and lung perfusion (E1). The VR is well correlated with the dead space fraction calculated by the Bohr equation (E2), and has the advantage of not requiring the measurement of expired carbon dioxide (CO_2_):

VR = (V_E_ x P_a_CO_2_) / (predicted V_E_ x ideal P_a_CO_2_)

Where V_E_ is the measured minute ventilation (in mL/min); P_a_CO_2_ is the arterial CO_2_ pressure (in mmHg); and predicted V_E_ is the predicted minute ventilation (in mL/min), corresponding to predicted body weight (kg) multiplied by 100. Lastly, the ideal P_a_CO_2_ is fixed at 37.5 mmHg (E2).

E1. Diehl J-L, Peron N, Chocron R, et al. Respiratory mechanics and gas exchanges in the early course of COVID-19 ARDS: a hypothesis-generating study. Ann Intensive Care 2020;10(1):95.

E2. Sinha P, Calfee CS, Beitler JR, et al. Physiologic Analysis and Clinical Performance of the Ventilatory Ratio in Acute Respiratory Distress Syndrome. Am J Respir Crit Care Med 2019;199(3):333–341.

**Additional file - Table E1**

The LRM and titration of the optimal PEEP.

| **Characteristics** | **n (%)**  **median [IQR]** |
| --- | --- |
| Maximum LRM, number of events (%) |  |
| Completed (PEEP = 40 cmH_2_O) | 20 (67) |
| Interrupted at PEEP = 35 cmH_2_O | 5 (17) |
| Interrupted at PEEP = 30 cmH_2_O | 4 (13) |
| Interrupted at PEEP = 25 cmH_2_O | 1 (3) |
| Reason for interrupting LRM, number of events (%) |  |
| Decrease in heart rate >20% | 3 (10) |
| Decrease in mean arterial pressure >20% | 9 (30) |
| Peripheral oxygen saturation <88% | 1 (3) |
| Optimal PEEP level, median [interquartile range], cmH_2_O | 12 [10-14] |

LRM: lung recruitment maneuver, PEEP: positive end-expiratory pressure.

**Additional file - Table E2**

Effects of LRM according to lung recruitability, in patients with positive end-expiratory transpulmonary pressure.

| **Parameters** | **R/I <0.62**  **n=11** | **R/I ≥0.62**  **n=11** | ***p* value** |
| --- | --- | --- | --- |
| P_a_O_2_/F_i_O_2_ ratio, mmHg | | | |
| P_a_O_2_/F_i_O_2_ pre-LRM | 116 [101-134] | 99 [71-131] | 0.566 |
| P_a_O_2_/F_i_O_2_ post-LRM | 187 [129-192] | 139 [98-171] | 0.713 |
| Δ P_a_O_2_/F_i_O_2_ (post-pre LRM) | 46 [10-91] | 41 [3-79] | 0.842 |
| *p* value | 0.027 | 0.045 |  |
| C_rs_, mL/cmH_2_O | | | |
| C_rs_ pre-LRM | 32 [22-41] | 42 [28-46] | 0.173 |
| C_rs_ post-LRM | 35 [23-43] | 44 [36-60] | 0.024 |
| Δ C_rs_ (post-pre LRM) | 4 [-1-6] | 8 [4-17] | 0.032 |
| *p* value | 0.240 | 0.018 |  |

C_rs_: respiratory system compliance, LRM: lung recruitment maneuver, P_a_O_2_/F_i_O_2_: partial pressure of oxygen to inspired oxygen fraction.

**Additional file - Table E3**

Hemodynamic changes in LRM according to lung recruitability.

| **Parameters** | **All patients**  **n=30** | **R/I <0.62**  **n=15** | **R/I ≥0.62**  **n=15** | ***p* value** |
| --- | --- | --- | --- | --- |
| Systolic blood pressure, mmHg |  |  |  |  |
| SBP pre-LRM | 123 [105-139] | 124 [115-142] | 115 [103-136] | 0.547 |
| SBP post-LRM | 114 [103-131] | 114 \|104-119] | 113 [104-134] | 0.868 |
| ΔSBP (post-pre LRM) | -6 [-24-3] | -8 [-22--2] | -5 [-24-6] | 0.350 |
| *p* value | 0.014 | 0.017 | 0.270 |  |
| Diastolic blood pressure, mmHg |  |  |  |  |
| DBP pre-LRM | 57 [51-69] | 54 [51-63] | 61 [55-71] | 0.135 |
| DBP post-LRM | 55 [51-61] | 54 [51-58] | 60 [51-64] | 0.299 |
| ΔDBP (post-pre LRM) | 0 [-7-3] | 0 [-4-4] | 0 [-8-1] | 0.560 |
| *p* value | 0.341 | 0.952 | 0.178 |  |

DBP: diastolic blood pressure, LRM: lung recruitment maneuver, SBP: systolic blood pressure.

**Additional file - Figure E1**

Protocol for the LRM in patient #12.

**Additional file - Figure E2**

Study flow chart.

**

**Additional file - Figure E3**

The receiver operating characteristic curve for the prediction of lung recruitment after the LRM based on the R/I ratio.
